# Supplementary material for: Hippocampal convergence during anticipatory midbrain activation promotes subsequent memory formation
Source: Nat Commun. 2022 Nov 7;13:6729. doi: 10.1038/s41467-022-34459-3 (PMC9640528; doi:10.1038/s41467-022-34459-3)

Hippocampal convergence during anticipatory midbrain activation promotes subsequent memory formation

Jia-Hou Poh[1], Mai-Anh T Vu[1,2,3], Jessica K Stanek[1,4], Abigail Hsiung[1,4], Tobias Egner[1,4], R. Alison Adcock[1,2,4,5]

[1] Center for Cognitive Neuroscience, Duke University, Durham NC, USA
[2] Department of Neurobiology, Duke University, Durham NC, USA
[3] Department of Psychological & Brain Sciences, Boston University, Boston, USA
[4] Department of Psychology & Neuroscience, Duke University, Durham NC, USA
[5] Department of Psychiatry & Behavioral Sciences, Duke University, Durham NC, USA

Correspondence:
R.Alison Adcock (alison.adcock@duke.edu) & Jia-Hou Poh (jiahou.poh@duke.edu)

Supplementary Information

Supplementary Results
Curiosity rating is a significant predictor of memory

For the primary analysis, we categorized trivia as ‘High’ or ‘Low’ curiosity based on the tertile split of each participant’s subjective rating. Here, we performed a mixed effects logistic regression using the curiosity rating as a continuous variable, and we showed that curiosity remained a significant predictor of memory (b = .48, SE = .034, p <.001).

To examine the possible influence of prior knowledge, we also included self-reported likelihood of knowing as a covariate. Curiosity remained a significant predictor of recall (b = .74, SE =.083, p <.001) after controlling for likelihood of knowing. However, it should be noted that our selection procedure entails the omission of trials with a high likelihood of knowing, and as such, was not designed to examine unique contributions of curiosity and prior knowledge.

Action-contingency was not a significant predictor of anticipatory activity across all ROIs

Control analyses with action-contingency as a predictor showed that there was no significant main effect nor interaction of action contingency across all ROIs (**VTA**: b = .038, SE = .045, p = .396 ; **HPC**: b = .050, SE = .041, p =.216; **PHC**: b = .048, SE = .042, p = .250; **PRC**: b = .055, SE = .036, p = .124; Supp Fig 3).

Relationship between VTA activity, hippocampal convergence and subsequent memory remained robust after controlling for delay interval

While the delay interval was not a primary variable of interest, we conducted several control analyses with delay interval included as a covariate to identify potential confounding effects. The additional analyses showed that all primary findings remained robust after controlling for delay. Curiosity remained a significant predictor of hippocampal convergence (b = -.017, SE = .007, p = .01), hippocampal convergence remained a significant predictor of subsequent memory (b = -.541, SE = .206, p = .008), and VTA remained a significant predictor of hippocampal convergence (b = -.058, SE = .003, p < .001).

Relationship between VTA activity, hippocampal convergence and subsequent memory remained robust after removing trials with outlying values

While including the mean amplitude as a covariate and using correlation distance measures reduces the influence of extreme values, we repeated key analyses after removing trials with outlying values for each subject (defined as trials with values exceeding 1.5 * interquartile range). A total of 15 outlying trials were identified, and the relationships remained significant between VTA activation and hippocampal typicality (b = -.048, SE = .011, p = .0003) and hippocampal typicality and memory (b = -.553, SE = .199, p = .005).

Model Specifications

Effect of curiosity on anticipatory activation

$BOLD_{ROI}$ ~ Curiosity + (1|Subject)

Effect of anticipatory activation on subsequent memory

Memory ~ $BOLD_{VTA}$ + $BOLD_{HPC}$ + $BOLD_{PHC}$ + $BOLD_{PRC}$ + (1|Subject)

Effect of curiosity on anticipatory pattern typicality

$Typicality_{ROI}$ ~ Curiosity + (1|Subject)

Effect of anticipatory pattern typicality on subsequent memory

Memory ~ $Typicality_{HPC}$ + $Typicality_{PHC}$ + $Typicality_{PRC}$ + (1|Subject)

Effect of VTA activation on anticipatory pattern typicality

$Typicality_{HPC}$ ~ $BOLD_{VTA}$ + (1+$BOLD_{VTA}$|Subject)

Supplementary Figure 1. Parameter estimates for the logistic regression of memory recall with univariate activation across both the Question and Answer intervals. Anticipatory BOLD activation (during both the Question and Answer interval) in the VTA and medial temporal lobe ROIs was used to predict memory outcome for each trial in a mixed-effects logistic regression model (N = 23 participants). This method allows the identification of variance that is uniquely accounted for by each of the ROIs across both intervals. During the Question interval VTA activation was the only statistically significant predictor of subsequent recall of answers (*p* =.015), while during the Answer interval PRC activation was the only statistically significant predictor of subsequent recall of answers (*p* = .003). Error bars represent the SEM. * $p < .05$, ** $p < .01$.

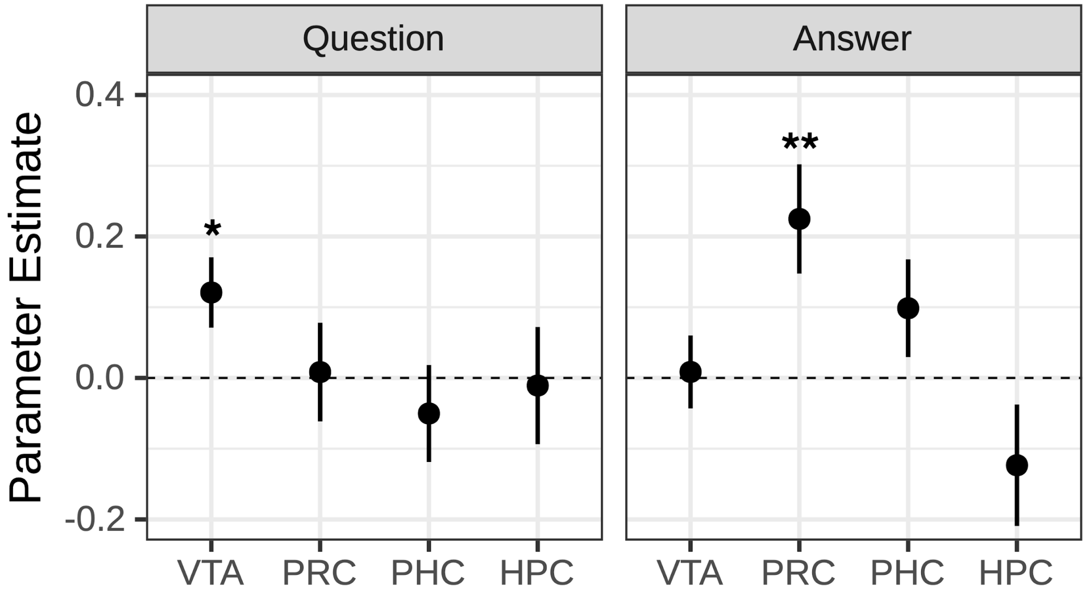

Supplementary Figure 2. State convergence in the medial temporal lobe during the presentation of answers. A) During the presentation of trivia answers, curiosity did not influence pattern typicality in any of the medial temporal lobe ROIs (N = 23 participants). The larger dots in each panel represents the group mean, while the smaller dots represent the mean distance for each participant. **B)** We used a mixed-effects logistic regression model to predict memory outcome for each trial using the pattern typicality of the medial temporal lobe ROIs (N = 23 participants). Pattern typicality in the medial temporal cortices (PHC & PRC), but not the Hippocampus, were significant predictors of subsequent recall. Bar graph of each panel represents the parameter estimate of each ROI in the full model. For visualisation, the estimated change in probability of recall (demeaned within subject) is plotted against the distance from centroid for each ROI. Light gray lines depict the slope for each participant, while the solid black line depicts the mean slope across all participants. Error bars represent the SEM. *** $p < .001$

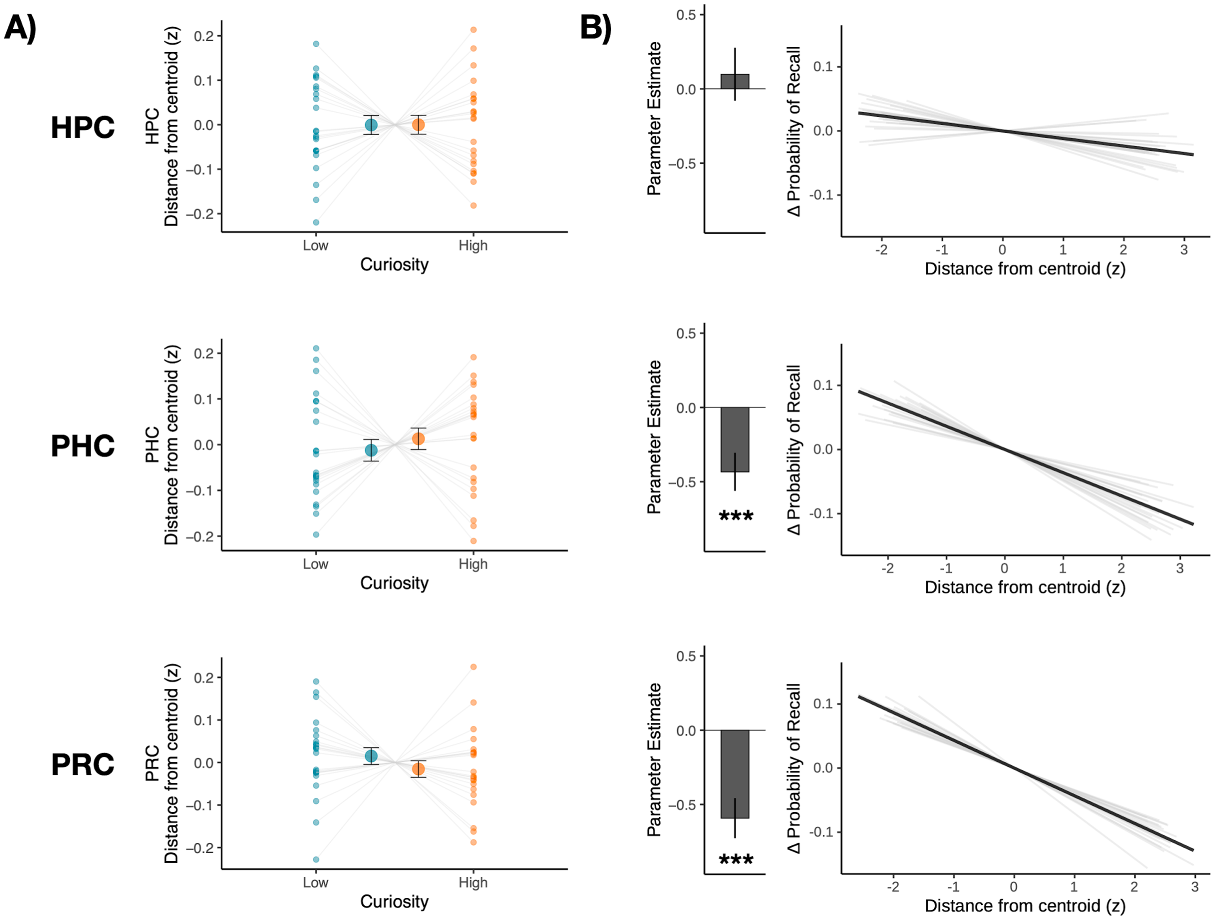

Supplementary Figure 3. Anticipatory activation following Question presentation across curiosity states and action-contingency. Across all ROIs, there was no significant effect of action-contingency on anticipatory activation (N = 23 participants). Red overlays on the brain images demarcate the ROIs (Brain template obtained from FSL and reproduced with permission). Error bars represent the SEM.

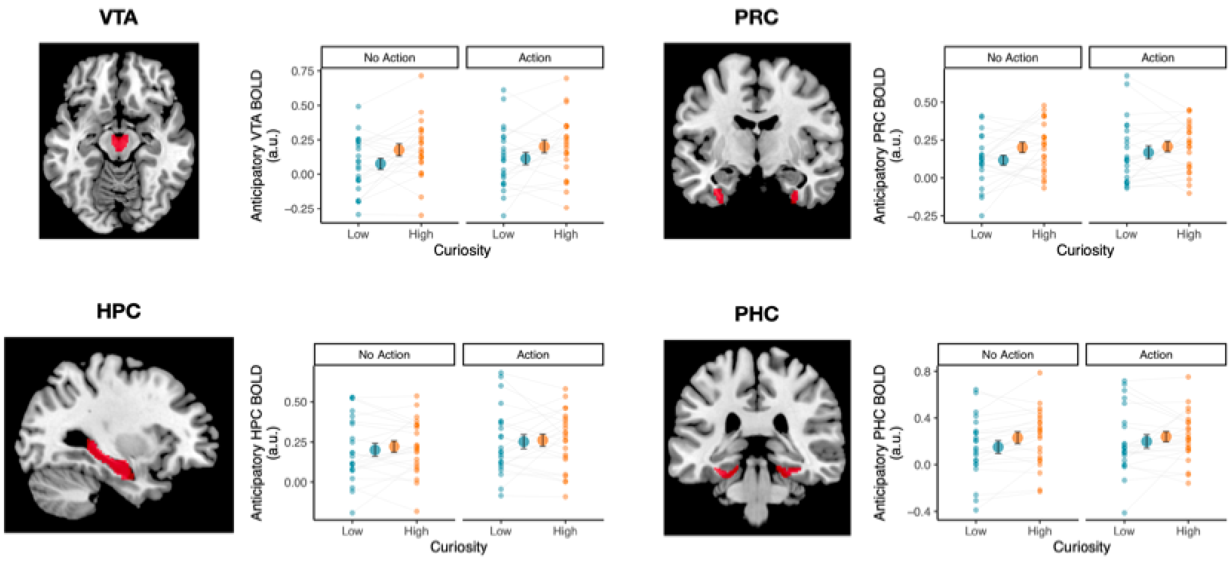

Supplementary Figure 4. Overlay of each medial temporal lobe region-of-interest on the normalised brain image of each subject. Red - Hippocampus; Blue - Parahippocampal cortex; Green - Perirhinal cortex. Template obtained from FSL and reproduced with permission.

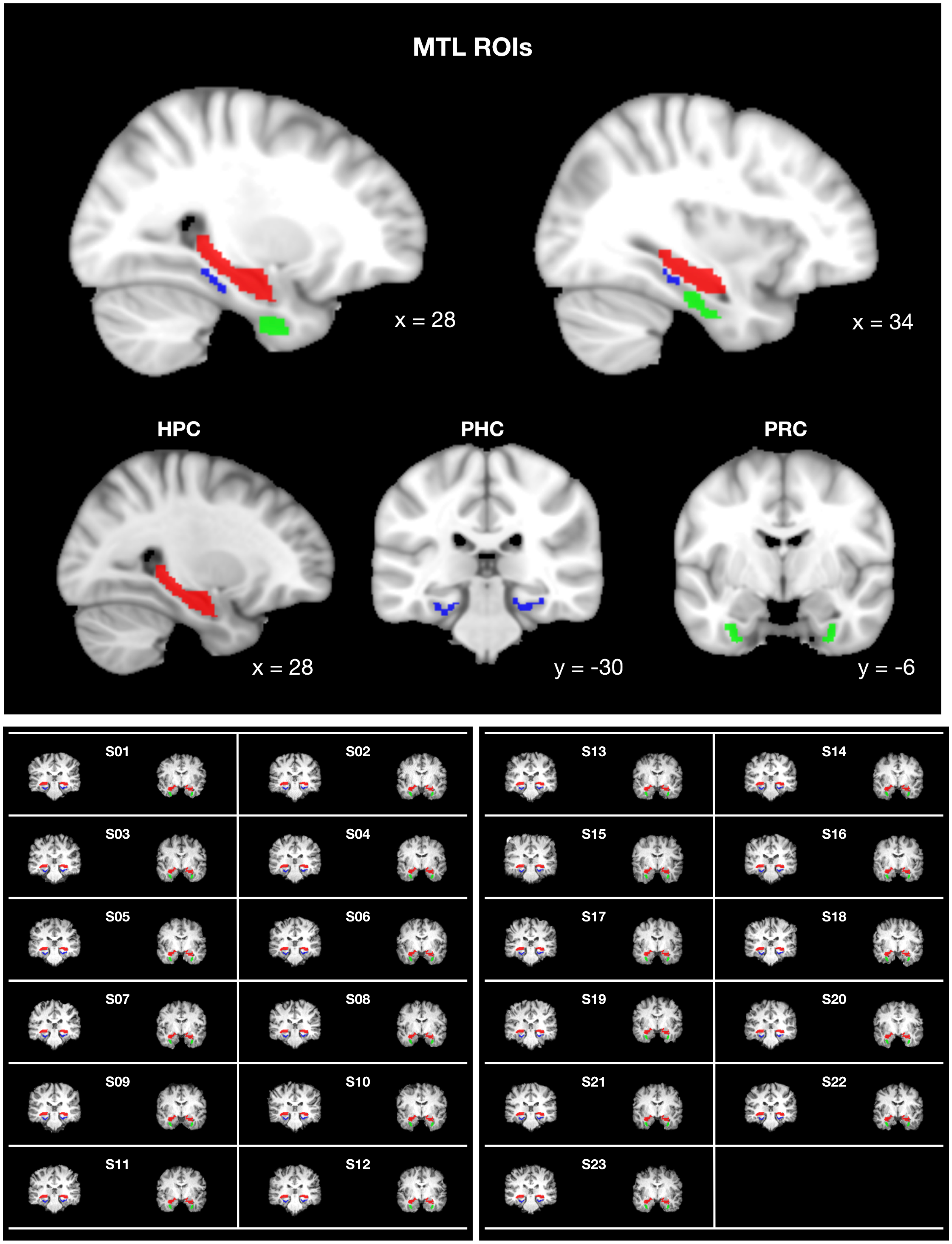

Supplement: Supplementary file 1 — Supplementary Information [file 41467_2022_34459_MOESM1_ESM.pdf]
