# Supplementary material for: Hippocampal convergence during anticipatory midbrain activation promotes subsequent memory formation
Source: Nat Commun. 2022 Nov 7;13:6729. doi: 10.1038/s41467-022-34459-3 (PMC9640528; doi:10.1038/s41467-022-34459-3)
Supplement: Supplementary file 3 — Reporting Summary [file 41467_2022_34459_MOESM3_ESM.pdf]

nature portfolio

Corresponding author(s): Jia-Hou Poh
Last updated by author(s): Aug 16, 2022

Reporting Summary

Nature Portfolio wishes to improve the reproducibility of the work that we publish. This form provides structure for consistency and transparency in reporting. For further information on Nature Portfolio policies, see our Editorial Policies and the Editorial Policy Checklist.

Statistics

For all statistical analyses, confirm that the following items are present in the figure legend, table legend, main text, or Methods section.

n/a	Confirmed	
☐	☒	The exact sample size (*n*) for each experimental group/condition, given as a discrete number and unit of measurement
☐	☒	A statement on whether measurements were taken from distinct samples or whether the same sample was measured repeatedly
☐	☒	The statistical test(s) used AND whether they are one- or two-sided<br>*Only common tests should be described solely by name; describe more complex techniques in the Methods section.*
☐	☒	A description of all covariates tested
☐	☒	A description of any assumptions or corrections, such as tests of normality and adjustment for multiple comparisons
☐	☒	A full description of the statistical parameters including central tendency (e.g. means) or other basic estimates (e.g. regression coefficient) AND variation (e.g. standard deviation) or associated estimates of uncertainty (e.g. confidence intervals)
☐	☒	For null hypothesis testing, the test statistic (e.g. *F*, *t*, *r*) with confidence intervals, effect sizes, degrees of freedom and *P* value noted<br>*Give P values as exact values whenever suitable.*
☒	☐	For Bayesian analysis, information on the choice of priors and Markov chain Monte Carlo settings
☒	☐	For hierarchical and complex designs, identification of the appropriate level for tests and full reporting of outcomes
☐	☒	Estimates of effect sizes (e.g. Cohen's *d*, Pearson's *r*), indicating how they were calculated

Our web collection on statistics for biologists contains articles on many of the points above.

Software and code

Policy information about availability of computer code

Data collection	Behavioral data was collected using Psychtoolbox implemented on Matlab. OptSeq2 was used for optimizing condition onset and trial intervals.
Data analysis	Statistical analysis was performed using linear and logistic mixed-effects modeling using the lme4 and lmerTest packages in R (Originally performed on Version 4.0.0, and reproduced on 4.2.0). Mediation analysis was conducted using the mediation package. Data visualization was created using ggplot2. Least-squares-all approach for fMRI analysis was implemented using FSL 5.0.8. Whole brain fMRI analysis was conducted using SPM12. Matlab code used for convergence analysis is available at - https://github.com/JiaHou-Poh/ConvergenceState. R markdown for reproducing the results is available at - https://github.com/JiaHou-Poh/TunedToLearn-AnticipatoryState.

For manuscripts utilizing custom algorithms or software that are central to the research but not yet described in published literature, software must be made available to editors and reviewers. We strongly encourage code deposition in a community repository (e.g. GitHub). See the Nature Portfolio guidelines for submitting code & software for further information.

Data

Policy information about availability of data

All manuscripts must include a data availability statement. This statement should provide the following information, where applicable:

- Accession codes, unique identifiers, or web links for publicly available datasets
- A description of any restrictions on data availability
- For clinical datasets or third party data, please ensure that the statement adheres to our policy

Source data are provided with this paper and are also available at - https://github.com/JiaHou-Poh/TunedToLearn-AnticipatoryState.

Human research participants

Policy information about studies involving human research participants and Sex and Gender in Research.

Reporting on sex and gender	23 Participants were included in the scan with 13 Male and 10 Female.
Population characteristics	All participants were healthy right-handed young adults (Mean age 26.4 years).
Recruitment	Participants were recruited through advertisement on various listservs and boards around Duke University and the Durham, NC community.
Ethics oversight	Duke Institutional Review Board.

Note that full information on the approval of the study protocol must also be provided in the manuscript.

Field-specific reporting

Please select the one below that is the best fit for your research. If you are not sure, read the appropriate sections before making your selection.

☒ Life sciences ☐ Behavioural & social sciences ☐ Ecological, evolutionary & environmental sciences

For a reference copy of the document with all sections, see nature.com/documents/nr-reporting-summary-flat.pdf

Life sciences study design

All studies must disclose on these points even when the disclosure is negative.

Sample size	Sample size was determined based on prior experiments using a similar experimental paradigm (e.g. Gruber et al., 2014).
Data exclusions	Due to a programming error, trivia questions for one participant were not correctly selected (based on screening), and 62 trials were removed from subsequent analysis. Two participants had to be excluded (one participant fell asleep during the scan, and one did not complete the scanning session).
Replication	Behavioral findings reported in the current study replicated findings from a previous study using a similar paradigm (Gruber et al., 2014). Effects of curiosity on memory has been observed in at least 3 other published studies (e.g. Kang et al., 2009; McGillivray et al., 2015; Stare et al., 2018) Neuroimaging findings reported are based on a single fMRI experiment, and the univariate findings observed in the current study replicated findings from Gruber et al., 2014. No further replication attempts have been made for the fMRI experiment.
Randomization	The study has a within-subject design and participants performed all conditions of the experiment. Stimuli used in the experiments are selected for each participant based on the reported pre-screening.
Blinding	No blinding was required. The study has a within-subject design and participants performed all conditions of the experiment.

Reporting for specific materials, systems and methods

We require information from authors about some types of materials, experimental systems and methods used in many studies. Here, indicate whether each material, system or method listed is relevant to your study. If you are not sure if a list item applies to your research, read the appropriate section before selecting a response.

Materials & experimental systems

n/a	Involved in the study
☒	☐ Antibodies
☒	☐ Eukaryotic cell lines
☒	☐ Palaeontology and archaeology
☒	☐ Animals and other organisms
☒	☐ Clinical data
☒	☐ Dual use research of concern

Methods

n/a	Involved in the study
☒	☐ ChIP-seq
☒	☐ Flow cytometry
☐	☒ MRI-based neuroimaging

Magnetic resonance imaging

Experimental design

Design type: Event-related task fMRI

Design specifications: Participants underwent a total of 6 scanning runs (10 mins each), with 12 high curiosity trials, 12 low curiosity trials, and 2 catch trials presented within each run. Task phase of interest (Question and Answer) within each trial was separated by 9 or 13s. Each trial was separated by an active baseline task that lasts between 1 to 20 seconds.

Behavioral performance measures: On trials that required a button press, a green arrow appeared on the left or right side of the screen, and participants made a button press to indicate the side that the arrow was presented on. Participants were highly accurate on the task (M = 98.6%, SD = 0.4). For the recall test, participants typed their response to a presented trivia question, and were allowed to skip the question if they were unable to remember the correct answer.

Acquisition

Imaging type(s): Functional MRI

Field strength: 3T

Sequence & imaging parameters: fMRI data for each participant were acquired using an echo-planar imaging (EPI) sequence (TE = 27ms, flip angle = 77 degrees, TR = 2000ms, voxel size = 3.75mm x 3.75mm) with 34 axial slices (slice thickness = 3.8mm).

Area of acquisition: Whole brain scan.

Diffusion MRI ☐ Used ☐ Not used

Preprocessing

Preprocessing software: Preprocessing of the fMRI data was performed using fMRI Expert Analysis Tool (FEAT) Version 6.00 implemented on FSL 5.0.8 (www.fmrib.ox.ac.uk/fsl). The first 6 volumes from each scan run were discarded to allow for signal stabilization. Physiological noise correction was performed using the Physiological Noise Modeling toolbox in FSL. Skull stripping was performed using BET, and images were realigned within-run, intensity normalized by a single multiplicative factor, spatially smoothed with a 4 mm full-width half-maximum (FWHM) kernel, and subjected to a high-pass filter (80s). The 4mm smoothing kernel was chosen to optimize the differentiation of midbrain and hippocampal signals

Normalization: Spatial normalization was performed using a two-step procedure, where mean EPI from each run was co-registered to the high-resolution anatomical image using FLIRT, which was followed by the normalization of the high-resolution anatomical image to MNI space using a nonlinear transformation with a 10mm warp resolution implemented with FNIRT.

Normalization template: MNI

Noise and artifact removal: Physiological noise correction was performed using the Physiological Noise Modeling toolbox in FSL..

Volume censoring: Motion parameters were included as regressors with no censoring performed.

Statistical modeling & inference

Model type and settings: Task modeling was performed using a least-square all approach and parameters were used for subsequent multivariate analyses.
All multivariate analyses were conducted using linear-mixed effects modeling with subjects included as random effects.
Exploratory whole brain voxel-wise analysis was conducted using mass univariate linear correlations.

Effect(s) tested: Memory performance was analyzed using a paired t-test comparing recall rates between the high and low curiosity condition. Univariate analyses were conducted using the mean value across all voxels within each ROI during the anticipation of trivia answers (following Question presentation). Linear-mixed effects analysis was conducted for each ROI with curiosity state as fixed effect, and subjects as random effect. A similar linear-mixed effects was conducted for the convergence analysis.

To examine if multivariate convergence in the hippocampus is associated with univariate activity in the midbrain VTA, a linear mixed effects model was implemented with trial-level univariate activation as a predictor of hippocampal convergence. The model included subjects as random intercepts, and VTA activity as a random slope. Mediation analysis was performed using the mediation package.

To examine the behavioral relevance of univariate and multivariate measures of brain activity, mixed effects logistic regression was performed with trial-level brain measures (i.e. univariate activation or multivariate state convergence) of all ROIs included as predictors of subsequent recall. By including all ROIs in a single model, this approach allows the identification of variance that is uniquely accounted for by the activity of each ROIs. For all mixed-effects models, subjects were included as random intercepts, and random slopes were included if it generated a better model fit based on model comparisons evaluated using a likelihood ratio test.

Specify type of analysis: ☐ Whole brain ☐ ROI-based ☒ Both

Anatomical location(s): To examine how activity in the midbrain interacts with the medial temporal lobe (MTL), we identified regions of interest which included the midbrain VTA and regions within the MTL. The VTA was defined using a probabilistic atlas thresholded at 50% 91. Three separate ROIs were defined within the medial temporal lobe, which included the hippocampus proper, perirhinal cortex and parahippocampal cortex. The hippocampus was defined using the AAL atlas, while the perirhinal and parahippocampal cortex were defined using anatomical mask from 92. All ROIs were defined in MNI space.

Statistic type for inference (See Eklund et al. 2016): Voxel-wise inference was used for the whole brain analysis.

Correction: Whole-brain analysis was thresholded using a permutation based approach with a 95th percentile thresholding and FWE corrected.

Models & analysis

n/a	Involved in the study
☒	☐ Functional and/or effective connectivity
☒	☐ Graph analysis
☐	☒ Multivariate modeling or predictive analysis

Multivariate modeling and predictive analysis: For the proposed analysis, patterns of activation in the ROIs are operationalised as points in an N-dimensional space, with N being the number of voxels in each ROI. Distance in the current analysis was measured using correlation distance (1 - Pearson's r), a distance metric commonly used in multivoxel pattern analysis. To examine the association between VTA activity and neural state in the medial temporal lobe, we devised an approach to quantify the trial-by-trial variation in neural state based on their distance from an independently defined centroid. The cluster centroid is a point with the shortest distance to all other points in high dimensional state space, and can be thought of as a protoypical state. We defined the centroid using a leave-one-run-out approach, where the cluster centroid was identified, with a k-means algorithm, using data from N-1 runs. This centroid was then used as the origin to quantify the distance for trials from the left-out run. Centroids for the analysis of the anticipatory period were defined using activation patterns from the Question interval, while analysis for the encoding of answers were defined using activation patterns from the Answer interval. This was repeated for all runs and was performed independently for each subject. The typicality for each trial was quantified based on their distance from the independently defined centroid. As the trials being measured do not contribute to the definition of the centroid (which they are measured relative to), this approach ensures the independence of the tested trials and the centroid-defining samples. Additionally, this also ensures that the quantification of typicality is not confounded by temporal correlation (since the centroid is defined using data from a different scanning run). In the current formulation, patterns closer to the centroid (i.e. shorter distance) are considered to exhibit greater convergence than patterns further from the centroid. This operationalization is similar to measures of neural variability, whereby a larger absolute difference from the average signal amplitude is considered to reflect greater trial-to-trial variability (e.g. 62). In contrast to a conventional linear classification approach, which would be suboptimal given the small number of datapoints and the imbalance between conditions in the current study (between number of Remembered and Forgotten trials), this approach also capitalizes on the expectation that successful memory formation is likely to require the confluence of multiple factors, and thus neural states conducive to memory formation should converge in state space. Linear-mixed effects analysis was conducted using the raw pattern typicality score for each ROI. For visualization, the measure of pattern typicality was z-scored across trials for each participant.
